# Epidemiological characteristics and spatiotemporal heterogeneity of hemorrhagic fever with renal syndrome in the Jiaodong Peninsula region, China, 2019–2025

**DOI:** 10.3389/fpubh.2026.1868358

**Published:** 2026-08-20

**Authors:** Xiaofang Guo, Ti Liu, Ruixiao Li, Xueying Tian, Yuwei Zhang, Xiaotong Shi, Qing Duan, Renpeng Li, Zengqiang Kou

**Affiliations:** 1 College of Public Health, Shandong Second Medical University, Weifang, China; 2 Shandong Center for Disease Control and Prevention, Jinan, China; 3 Shandong Provincial Key Laboratory of Intelligent Monitoring, Early Warning, Prevention and Control for Infectious Diseases, Jinan, China; 4 Shandong Mental Health Center, Shandong University, Jinan, China

**Keywords:** epidemiological characteristics, hemorrhagic fever with renal syndrome, joinpoint regression analysis, spatial autocorrelation, spatiotemporal clustering, spatio-temporal geographically weighted regression model

## Abstract

**Background:**

Hemorrhagic fever with renal syndrome (HFRS) is a serious zoonotic infectious disease. To characterize the spatiotemporal epidemiological patterns of HFRS in the Jiaodong Peninsula from 2019 to 2025 and to explore the influence of meteorological factors on its spatiotemporal heterogeneity, the present study conducted relevant analyses with the aim of providing evidence for the development of scientific and effective HFRS prevention and control measures.

**Method:**

Data on HFRS were collected through the Chinese Center for Disease Control and Prevention Information System. Descriptive epidemiology, Joinpoint regression analysis, spatial autocorrelation analysis, spatiotemporal clustering analysis and spatiotemporal geographically weighted regression model analysis were employed to examine the incidence of HFRS in the Jiaodong Peninsula region from 2019 to 2025.

**Result:**

From 2019 to 2025, a total of 1,492 cases of HFRS were reported in the Jiaodong Peninsula region, with an average annual incidence rate of 1.073 per 100,000 people. The peak incidence period occurs annually from October to January of the following year. The age distribution is concentrated among those aged 45–74, with a male-to-female ratio of 2.58:1, and the occupational distribution is dominated by farmers. Overall, the spatial distribution pattern shows higher rates in the north and west compared to the south and east. Analysis using a join-point regression model indicates that the incidence rate showed an upward trend from 2019 to 2021, with an annual percentage change (APC) of 40.70% (95% CI: −15.72 to 134.88%, *p* = 0.103); however, this upward trend was not statistically significant. The incidence rate showed a downward trend from 2021 to 2025, with an APC of −13.60% (95% CI: −25.55 to 0.26%, *p* = 0.052); this downward trend was marginally significant. Analysis of global spatial autocorrelation revealed that, with the exception of 2019 (Moran’s I = 0.205, *p* = 0.052) and 2022 (Moran’s I = 0.189, *p* = 0.059), the incidence of HFRS showed spatial clustering in all other years (all with *p* < 0.05). Among these, 2021 had the highest agglomeration index (Moran’s I = 0.438). Analysis of local spatial autocorrelation shows that “high-high” clusters are primarily concentrated in the central and western parts of Yantai (around Laiyang, Haiyang, and Laizhou) and in the northern part of Qingdao (around Laixi). Spatio-temporal scanning analysis identified a total of three clusters, all concentrated in the fall and winter of 2021. The largest cluster spanned seven counties (cities and districts), primarily distributed across the inland and coastal areas of central and western Yantai (Laizhou, Zhaoyuan, Longkou, Laiyang, and Qixia) and northern Qingdao (Pingdu and Laixi). The Core Cluster scanning radius was 80.54 km. The high-incidence period was from November 1, 2021, to December 31, 2021. The Relative risk (RR) = 13.56, Log-likelihood ratio ((LLR) = 194.16, *p* < 0.01). GTWR model results demonstrated significant spatiotemporal heterogeneity of meteorological impacts on HFRS. Temperature showed an overall positive effect with a spatial pattern of west-strong and east-weak, south-high and north-low. Precipitation was positively associated with HFRS in eastern study areas but negatively in western areas, while relative humidity showed the opposite spatial pattern. Total solar radiation and wind speed exerted consistent negative inhibitory effects across the whole region, with wind speed presenting the strongest suppression. Atmospheric pressure mainly played a negative role, with only weak positive effects in partial southern Qingdao.

**Conclusion:**

From 2019 to 2025, HFRS in the Jiaodong Peninsula region exhibited characteristics of seasonal prevalence, spatiotemporal clustering, and spatiotemporal heterogeneity. Health education should be strengthened among key populations, such as middle-aged and older male farmers, and prevention and control measures should be intensified in high-risk areas, including central-western Yantai and northern Qingdao. Future prevention and control strategies should incorporate region-specific climatic characteristics to formulate targeted interventions.

## 1 Introduction

Hemorrhagic fever with renal syndrome (HFRS) is an acute zoonotic disease caused by hantaviruses, which belong to the Hantaviridae family. It is primarily transmitted by rodents (such as the black-striped field mouse and the brown rat), among these, Hantaan virus (HTNV) and Seoul virus (SEOV) are the predominant circulating strains of HFRS in China (1, 2). This disease is characterized by fever, bleeding, and acute kidney injury. In severe cases, it can lead to life-threatening conditions such as hypotensive shock and multiple organ dysfunction, posing a serious threat to human health. It has been classified as a Category B infectious disease requiring priority prevention and control in China (3–5). China bears the heaviest global burden of HFRS, with reported cases accounting for approximately 70–90% of the global total (6). Since the HFRS vaccine was incorporated into the National Expanded Program on Immunization in 2008, the national incidence rate has shown a downward trend, however, recent surveillance data indicate that the affected areas continue to expand (7). Shandong Province is one of the provinces in China with a historically high incidence of HFRS, accounting for approximately one-third of the nation’s total reported cases. The epidemic situation remains severe, placing sustained pressure on regional public health prevention and control efforts (8, 9).

The Jiaodong Peninsula region is located in the eastern part of the Shandong Peninsula and encompasses all counties (cities and districts) within Yantai, Qingdao, and Weihai. Surrounded by the sea on three sides and characterized by a predominantly hilly terrain, the region features a humid climate with distinct seasons. With its intermingled urban and rural areas and dense population, it provides suitable conditions for the proliferation of Hantavirus reservoir animals such as the black-striped field mouse and the brown rat, making it one of the traditional high-incidence areas for HFRS in Shandong Province (10). Meteorological factors may play a significant role in the transmission of HFRS. As demonstrated by Bao et al., the incidence of HFRS is likely influenced by a combination of complex factors, such as environmental and climatic conditions, rather than a single causative factor (11, 12). Currently, there is a lack of systematic research on the spatiotemporal epidemiological patterns of HFRS and the identification of high-risk clusters across the entire Jiaodong Peninsula region, covering all counties (cities and districts) in the three cities. Furthermore, no studies have yet used a spatio-temporally geographically weighted regression (GTWR) model to quantify the effects of spatio-temporal heterogeneity in meteorological factors (13). ArcGIS software has been widely used for visualizing the geographic distribution of epidemics and analyzing spatial patterns. SaTScan software can effectively detect spatiotemporal clusters of disease, identifying the scope and time periods of high-risk clusters, while Joinpoint regression analysis can accurately identify inflection points and rates of change in long-term trends of incidence. The combined application of these three methods can comprehensively and systematically reveal the epidemiological distribution patterns of HFRS, accurately identify high-risk areas and key populations, and provide a scientific basis for formulating differentiated, tiered prevention and control strategies (14, 15). The GTWR model quantifies the spatio-temporally non-stationary characteristics of meteorological factors influencing HFRS incidence. It accurately identifies variations in the intensity of these factors across different counties (cities, districts) within the same time period and across different years within the same region. The model provides an in-depth explanation of the mechanisms underlying HFRS incidence and offers data support for the development of refined, region-specific prevention and control strategies.

This study is based on HFRS case surveillance data from various counties (cities and districts) in the Jiaodong Peninsula region from 2019 to 2025. It employs descriptive epidemiological methods to analyze the spatial, temporal, and population-based distribution characteristics of HFRS, and utilizes R, ArcGIS, SaTScan, and Joinpoint software to analyze long-term trends in HFRS incidence. We further employed the GTWR model to quantify the spatiotemporal non-stationarity of meteorological factors on the incidence of HFRS, and to analyze differences in the intensity of these factors across different years and counties (cities, districts). The study aims to systematically elucidate the spatiotemporal epidemiological characteristics and clustering patterns of HFRS, accurately identify high-risk areas and time periods, and conduct an in-depth investigation into the spatiotemporal mechanisms underlying HFRS incidence, thereby filling the gap in fine-scale spatiotemporal epidemiological research in the Jiaodong Peninsula region. It will provide a scientific basis for formulating strategies such as regional HFRS surveillance and early warning, interventions targeting key populations, and rodent control measures, and holds significant public health implications for reducing the disease burden and enhancing public health prevention and control capabilities.

## 2 Materials and methods

### 2.1 Study area

**Figure 1:**
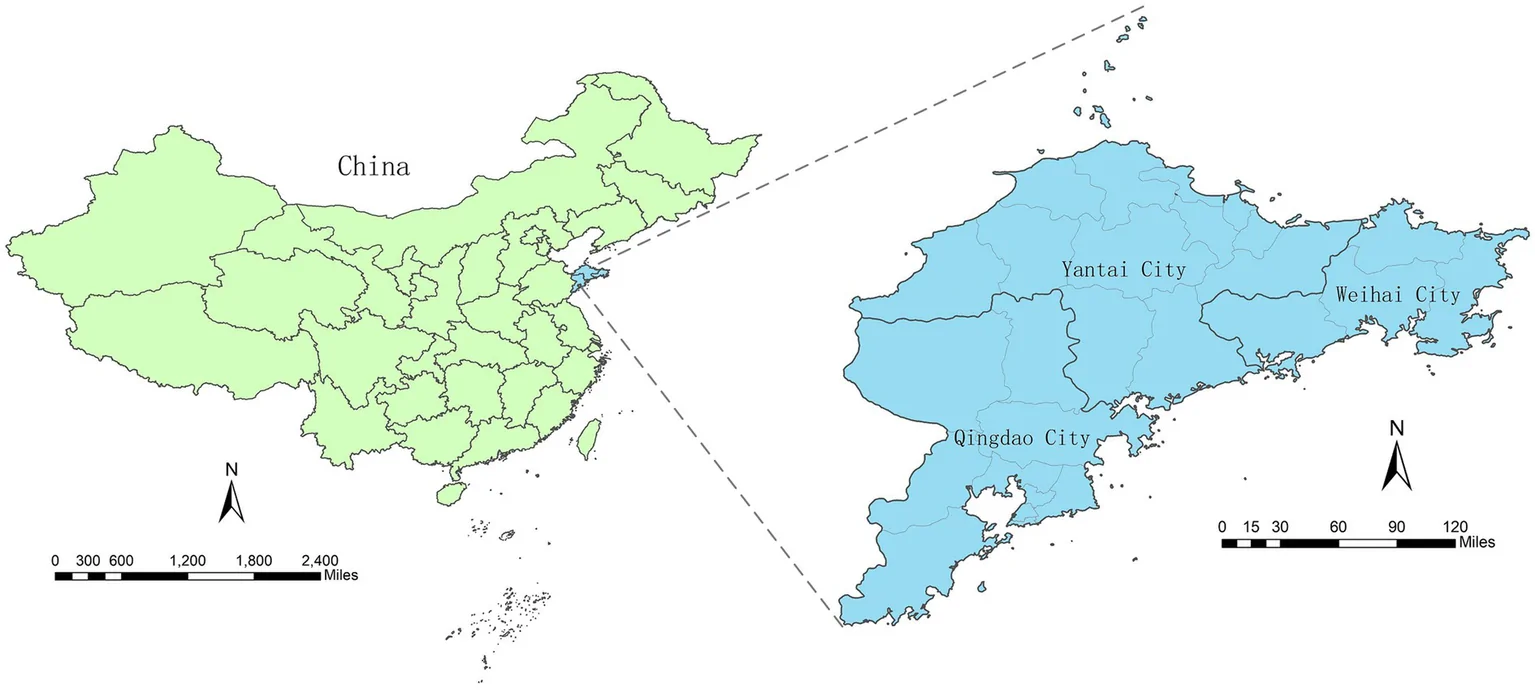
Geographic location of China’s Jiaodong Peninsula, 2019–2025.

The Jiaodong Peninsula is located in eastern Shandong Province, east of the Jiaolai River, at 35°35′–38°23′ north latitude and 119°30′–122°42′ east longitude. It is surrounded by the sea on the east, south, and north, and faces the Liaodong Peninsula across the Bohai Strait to the north and across the Yellow Sea from the Korean Peninsula to the east. It is China’s largest peninsula and a key geographical unit in the Bohai Rim region (See Figure 1). The study area consists primarily of the three cities of Qingdao, Yantai, and Weihai, with a total area of approximately 30,000 km^2^, the landscape is dominated by low mountains and hills, interspersed with river valley plains and coastal basins, and features a long and winding coastline (16).

### 2.2 Data sources

Collect all cases within the region from January 1, 2019, to December 31, 2025, from the Infectious Disease Surveillance System of the Chinese Center for Disease Control and Prevention (China CDC) Information System, including confirmed cases, suspected cases, and clinically diagnosed cases. The diagnostic criteria for HFRS are based on the *Expert Consensus on the Prevention and Treatment of Hemorrhagic Fever with Renal Syndrome (2021 Edition)* (17). Basic case information includes age, gender, occupation, address, and date of onset. Collect annual data on the permanent resident population from the Basic Information System of the China CDC Information System. Based on the database ERA5 (European Centre for Medium-Range Weather Forecasts Reanalysis Version 5) monthly averaged data on single levels from 1979 to present, collect annual meteorological data for 27 counties (cities and districts) in the Jiaodong Peninsula region from January 1, 2019, to December 31, 2025, including meteorological factors such as temperature, air pressure, relative humidity, wind speed, and precipitation. To ensure the comparability of spatiotemporal analyses for the 2019–2025 period, this study standardized the administrative divisions of counties (cities and districts) in the Jiaodong Peninsula during the data preparation phase. Statistics were calculated using 27 consistent administrative units throughout the study, thereby eliminating inconsistencies in spatial units caused by boundary adjustments-such as the conversion of counties into districts and the merger of townships-that occurred during the study period. Additionally, this study employs incidence rates for analysis, using the permanent resident population as the denominator to eliminate the confounding effects of population size differences across administrative units.

### 2.3 Statistical methods

#### 2.3.1 Descriptive analysis

Collect and compile data on the number of HFRS cases reported annually by county (city, district) in the Jiaodong Peninsula region. Using the permanent resident population data for each county (city, district) in the Jiaodong Peninsula region for the corresponding year as the denominator, and the total number of newly confirmed HFRS cases in that county (city, district) for that year as the numerator, the crude incidence rate of HFRS (per 100,000) is calculated by year and by region, and compile case statistics by age, gender, and occupation, and establish a research database. Descriptive epidemiological analysis of the data was performed using R version 4.5.2. The Joinpoint software was used to assess the temporal trends in the overall incidence of HFRS. Using Python software to analyze ordinary least squares (OLS) regression, geographically weighted regression (GWR), and the GTWR model, this study investigates the spatio-temporal heterogeneity of HFRS incidence based on meteorological data. ArcGIS 10.8.1 was used to map the spatial distribution of cases and local regression coefficient maps to complete the spatial visualization. The SaTScan software was used to identify clusters.

#### 2.3.2 Joinpoint regression analysis

The Joinpoint regression model was used to analyze the temporal characteristics of HFRS incidence trends in the Jiaodong Peninsula region from 2019 to 2025. This model uses the grid search method to automatically identify joinpoints where significant changes in trends occur during this period, dividing the long-term trend into several segments. It calculates the annual percentage change (APC) for each segment and the average annual percentage change (AAPC) for the entire period to analyze the rate and direction of trend changes across different time periods, and assess the development trends of HFRS (18, 19). Regression models were constructed using Joinpoint 5.1.0 software, with year as the independent variable and incidence rate as the dependent variable, the minimum number of observations was set to 2, and the maximum number of breakpoints to 1. Based on the distribution characteristics of the HFRS incidence data, a log-linear model (based on the Poisson distribution) was used for fitting. A Monte Carlo permutation test was used to test the statistical significance of the annual percentage change (APC) and inflection points for each time period, with a significance level of *α* = 0.05. An APC or AAPC > 0 indicates an upward trend in the HFRS incidence rate for that time period, while a value less than 0 indicates a downward trend. The formula for the Joinpoint regression model is:


$$y=\{\begin{array}{ll} a_{1}+b_{1}x, & x\leq \tau_{1}\\ a_{2}+b_{2}x, & \tau_{1}<x\leq \tau_{2}\\ \;\vdots & \\ a_{K+1}+b_{K+1}x, & x>\tau_{K} \end{array}$$

Among these, x represents time, y represents incidence, τ represents the connection point, b represents the slope of each segment, and a represents the y-intercept of each segment.

#### 2.3.3 Spatial autocorrelation analysis

Using the ArcToolbox in ArcGIS 10.8.1, we conducted spatial autocorrelation analyses of HFRS case counts for each county (city, district) in the Jiaodong Peninsula region from 2019 to 2025, including both global and local spatial autocorrelation analyses. Global spatial autocorrelation is assessed using Moran’s I to evaluate whether HFRS cases are spatially clustered. Moran’s I ranges from −1 to 1. A value of Moran’s I > 0 indicates positive spatial correlation (the closer the value is to 1, the stronger the spatial correlation and the more significant the clustering), while a value of Moran’s I < 0 indicates negative spatial correlation (the closer the value is to −1, the stronger the negative spatial correlation and the greater the variation). Moran’s I = 0 indicates a random distribution with no spatial correlation. Statistical significance is tested using the Z-test. A difference is considered statistically significant when |Z| > 1.96 (i.e., Z > 1.96 or Z < −1.96) and *p* < 0.05, indicating the presence of significant spatial autocorrelation (20). Local Spatial Autocorrelation (LISA) uses Anselin’s Local Moran’s I statistic to identify specific areas of HFRS clustering. Clustering patterns are classified into four types: high-high clusters (H-H, hotspots), low-low clusters (L-L, coldspots), high-low clusters (H-L), and low-high clusters (L-H, outliers). Hypothesis testing of the LISA statistic was performed using the Z-test (*p* < 0.05 indicates statistical significance). Finally, the spatial clustering patterns of HFRS in the Jiaodong Peninsula region were visualized using ArcGIS 10.8.1 software (21).

#### 2.3.4 Spatio-temporal clustering analysis

Using SaTScan v9.7 software, we conducted a spatio-temporal clustering analysis of HFRS cases in each county (city, district) of the Jiaodong Peninsula from 2019 to 2025. The time range was set from January 1, 2019, to December 31, 2025, with a time interval of “month.” The maximum spatial scanning radius was set at 30% of the total population in the study area, and the temporal window was set at 30% of the study period. Based on the distribution characteristics of HFRS cases, a Poisson distribution model was selected for analysis. Significance tests were performed using Monte Carlo simulation (999 iterations), and the strength of disease clustering was assessed by calculating the log-likelihood ratio (LLR). When the difference in LLR was statistically significant (*p* < 0.05), the region was considered to exhibit clustering (22). Clusters with the highest LLR values and p < 0.05 were classified as Type 1 cluster, while the remaining statistically significant clusters were classified as Type 2 and Type 3 cluster, respectively. Finally, the results of the spatiotemporal clustering analysis were visualized using ArcGIS 10.8.1.

#### 2.3.5 Spatio-temporal geographically weighted regression analysis

Python software was used to conduct geographic and time-weighted regression (GTWR) model analysis, examining the spatio-temporal heterogeneity of risk factors for HFRS cases in each county (city, district) of the Jiaodong Peninsula from 2019 to 2025. The annual incidence rate in each county (city, district) was used as the dependent variable, and multiple meteorological indicators-including air temperature, atmospheric pressure, and precipitation-were selected as independent variables and included in the model. A weight matrix was constructed based on spatiotemporal distance, the Bi-square kernel function was used to model spatiotemporal proximity effects, outliers were removed using the interquartile range method, and independent variables were standardized using Z-score standardization (standard deviation standardization). The adaptive bandwidth was optimized using the Akaike Information Criterion (AICc), local regression equations were fitted to obtain the influence coefficients for each spatio-temporal cell, and significance tests were performed on the local regression coefficients (*p* < 0.05). At the same time, ordinary least squares (OLS) and geographically weighted regression (GWR) models were constructed for comparison. The models were evaluated based on metrics such as the coefficient of determination (R^2^), adjusted R^2^, and AICc. This allowed us to quantify the strength and direction of the influence of meteorological factors on disease incidence levels across different years and counties. Spatial visualizations of the local regression coefficients for each factor were generated using ArcGIS 10.8.1 software. Building upon the traditional geographically weighted regression (GWR) model (13, 23). Huang et al. developed and optimized the spatio-temporal geographically weighted regression (GTWR) model. This model incorporates a spatio-temporally non-stationary weight matrix, by calculating the spatio-temporal distances within the study area, it constructs a continuous distance decay function. The spatio-temporal coordinates of each observation unit and the corresponding variable values are then substituted into the decay function to determine the weight coefficients, thereby deriving the weighted regression equation (24). The formula for the GTWR regression model is:


$$y_{i}=\beta_{0}(u_{i},\nu_{i},t_{i})+\sum_{k=1}^{k}\beta_{k}(u_{i},\nu_{i},t_{i})X_{\mathrm{ik}}+\varepsilon_{i}$$

Here, i = 1, 2, 3, …, n. For each observation i, y_i_ is the dependent variable, and X_ik_ is the kth explanatory variable (u_i_,v_i_,t_i_) denotes the spatiotemporal coordinates of observation i, u_i_ and v_i_ are the projected spatial coordinates, and t_i_ is the projected temporal coordinate, β_0_(u_i_,v_i_,t_i_) is the intercept term, β_0_(u_i_,v_i_,t_i_) is the kth regression coefficient for the i-th data point. εᵢ is the constant term, representing the random error at observation i. It is assumed to have a mean of 0 and a variance of σ^2^ = 0.

## 3 Results

### 3.1 Overview

**Figure 2:**
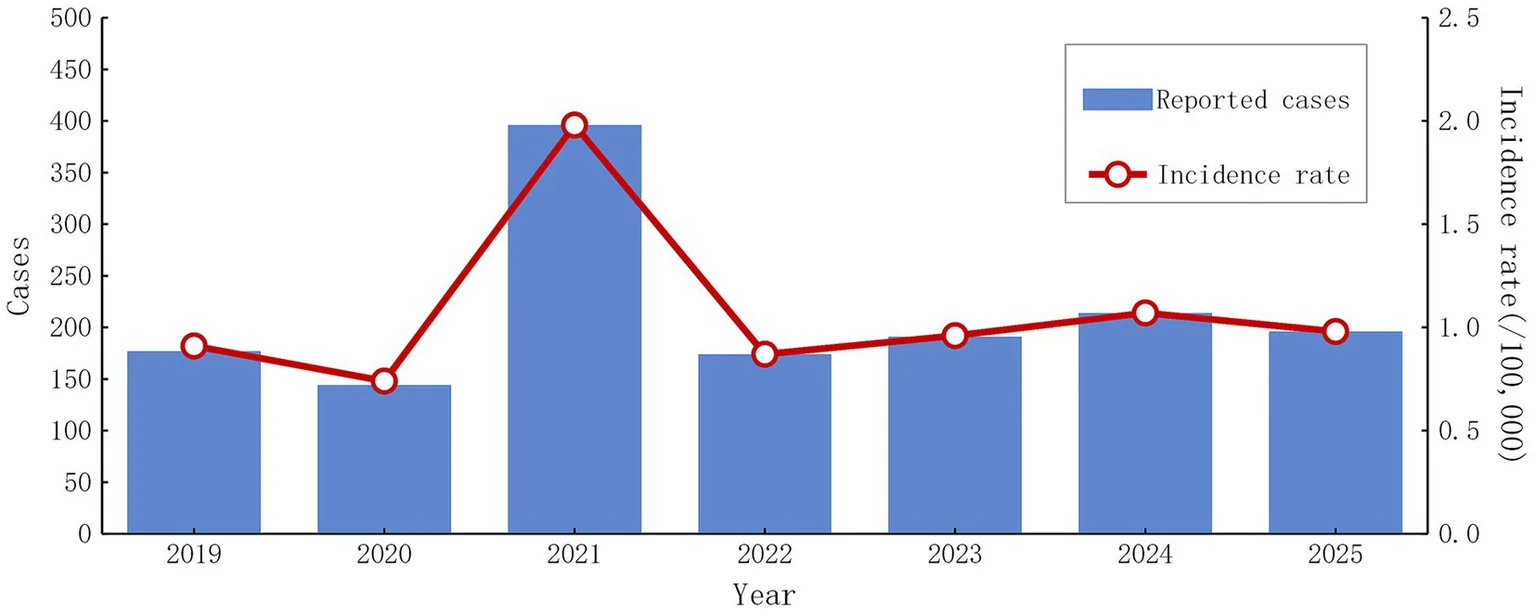
Number of HFRS cases reported and incidence rates in the Jiaodong Peninsula Region, 2019–2025.

From 2019 to 2025, a total of 1,492 cases of hemorrhagic fever with renal syndrome (HFRS) were reported in the Jiaodong Peninsula region, with an average annual incidence rate of 1.073 per 100,000 people. Of these, Qingdao reported 796 cases (53.4%), Yantai reported 565 cases (37.9%), and Weihai reported 131 cases (8.7%). An analysis of incidence trends shows that the incidence of HFRS has fluctuated over time, rising from 0.915 per 100,000 in 2019 to 1.981 per 100,000 in 2021, with a particularly significant increase observed between 2020 and 2021. After peaking in 2021, the incidence rate declined rapidly, from 2022 to 2025, it remained between 0.873 and 1.071 per 100,000, fluctuating slightly, with the epidemic tending toward relative stability (see Figure 2).

### 3.2 Characteristics of epidemic distribution

#### 3.2.1 Temporal distribution

**Figure 3:**
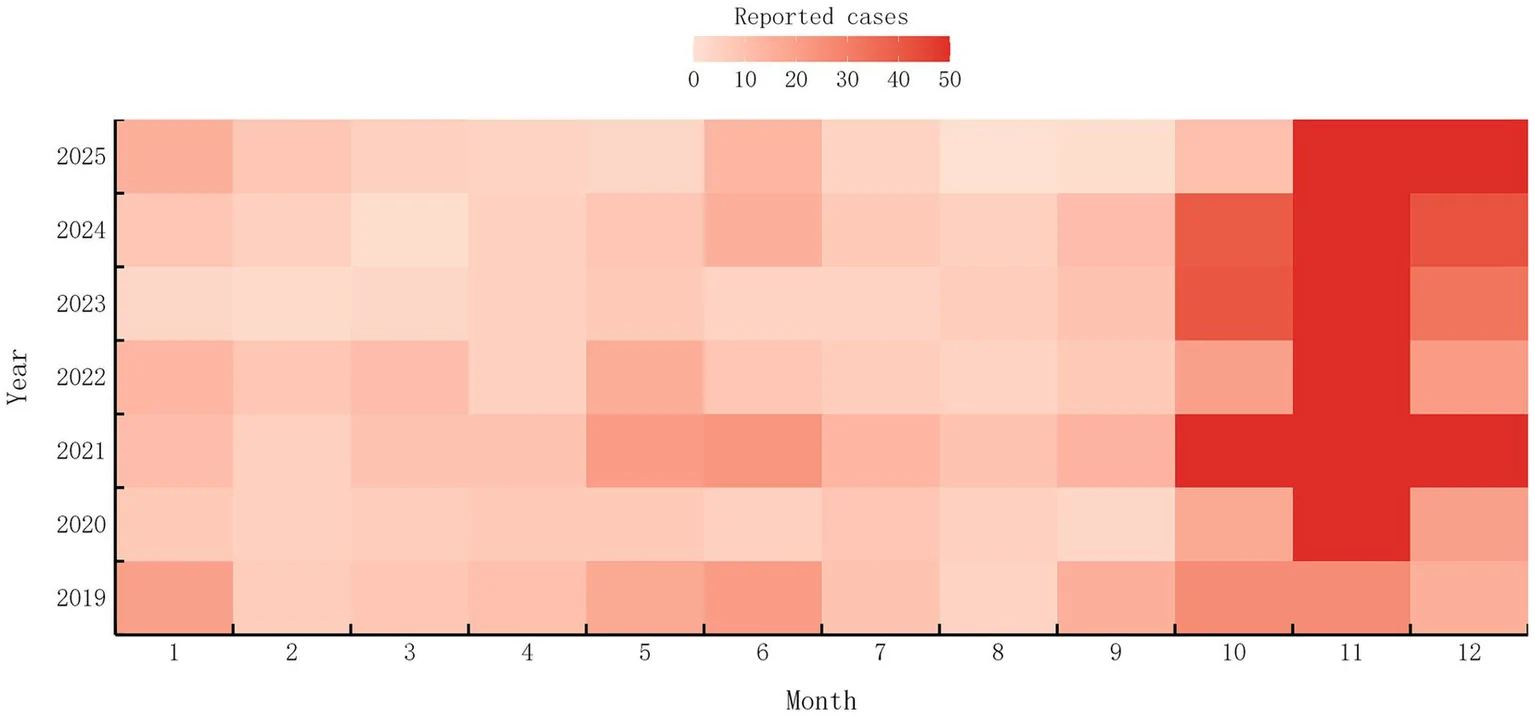
Monthly distribution heatmap of HFRS in the Jiaodong Peninsula region, 2019–2025.

From 2019 to 2025, HFRS incidence in the Jiaodong Peninsula region exhibited a distinct seasonal pattern. The seasonal patterns were highly consistent across all years, showing a bimodal distribution: a peak in late spring to early summer (May–June) and a peak in autumn and winter (October–January of the following year), with the autumn and winter peak being the predominant one. During the autumn and winter peaks from 2019 to 2025 (October through January of the following year), a cumulative total of 1,053 cases were reported, accounting for 70.58% of the total number of cases, making this period a critical time for HFRS prevention and control. Among these, the number of cases peaked in November 2021 (147 cases), marking the highest monthly reported case count between 2019 and 2025, which aligns with the timing of the outbreak that year (See Figure 3).

#### 3.2.2 Geographic distribution

**Figure 4:**
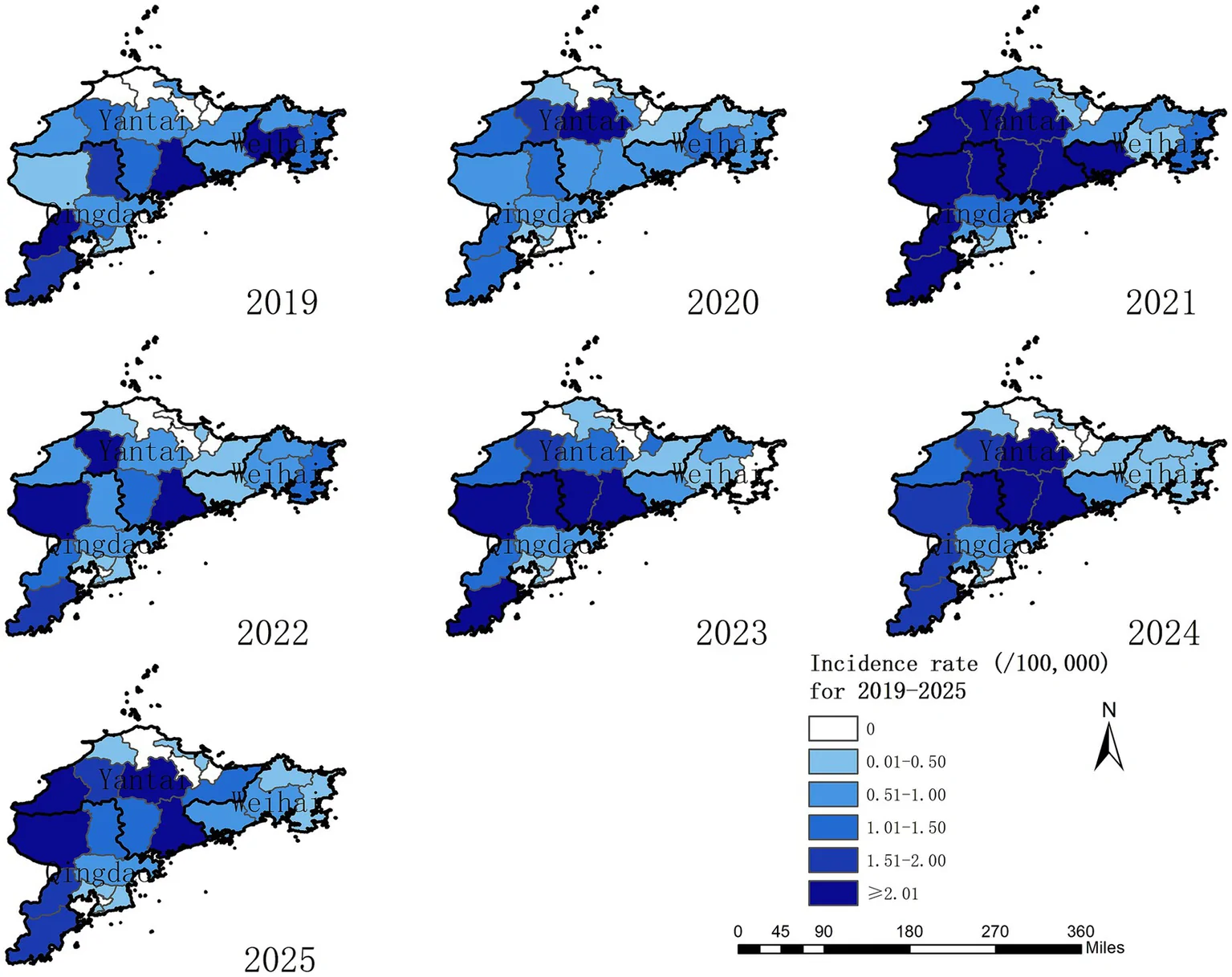
Map of the distribution of counties (districts, cities) by HFRS incidence rate in the Jiaodong Peninsula region, 2019–2025.

Among the 27 counties (cities and districts) in the Jiaodong Peninsula region from 2019 to 2025, the number of counties and districts covered by HFRS reports increased from 21 in 2019 to 25 in 2021, indicating a continuing trend of expansion in the affected areas. The top six counties (cities and districts) with the highest cumulative number of reported cases are, in order: Huangdao District (261 cases), Pingdu City (174 cases), Haiyang City (153 cases), Jiaozhou City (119 cases), Laiyang City (98 cases), and Laizhou City (92 cases). No cases have been reported in Changdao County, Yantai. Areas with high incidence rates of HFRS are primarily concentrated in central Yantai (the Laiyang, Haiyang, and Qixia regions) and western Yantai (Laizhou), as well as in northwestern Qingdao (the Pingdu, Laixi, Jiaozhou, and Huangdao regions) and southern Weihai (the Rushan region). Overall, the spatial distribution pattern shows higher incidence rates in the north and west compared to the south and east (See Figure 4).

#### 3.2.3 Population distribution

**Figure 5:**
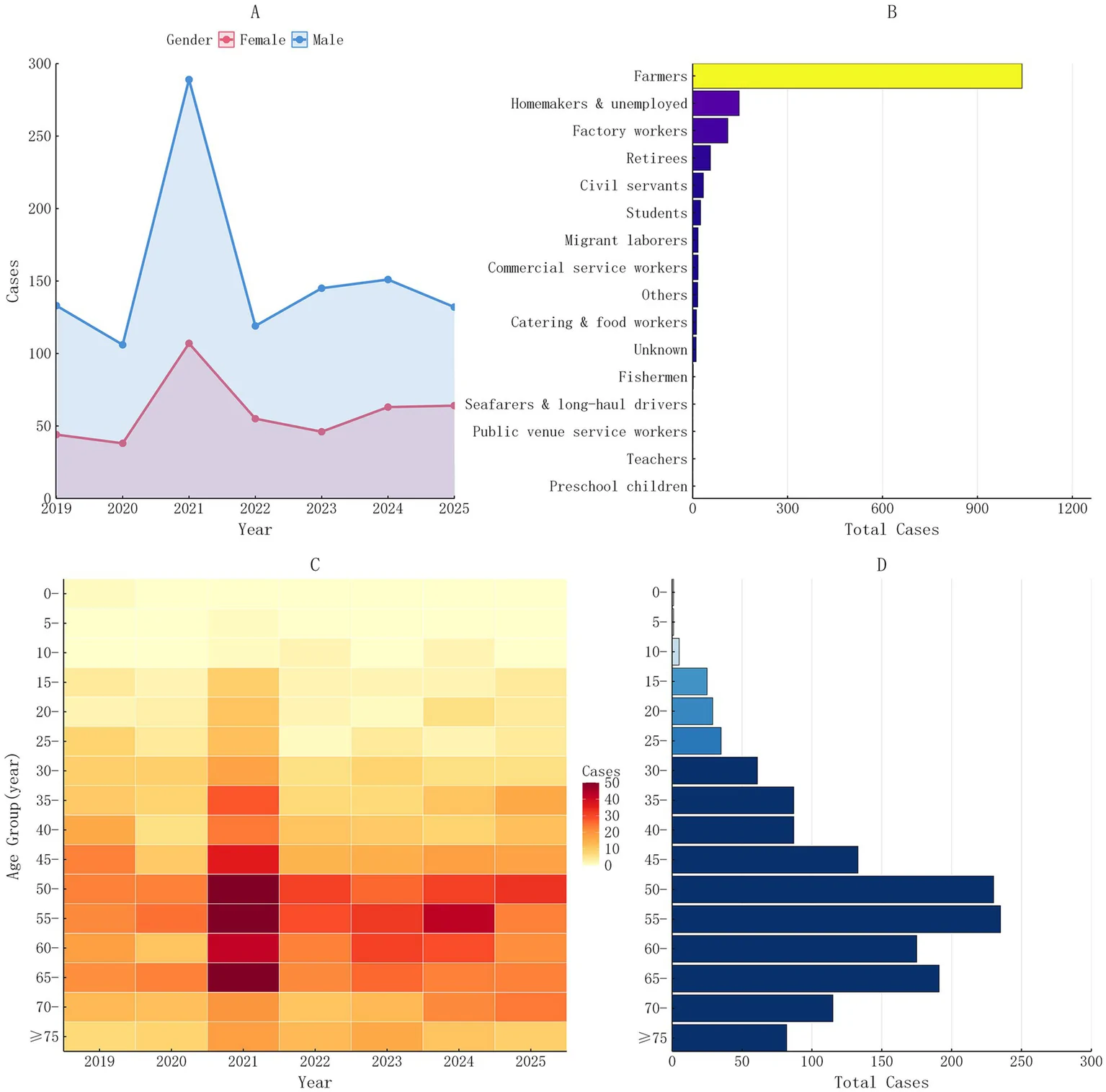
Distribution of demographic characteristics of HFRS cases reported in the Jiaodong Peninsula Region, 2019–2025. **(A)** Line chart showing reported HFRS cases by gender in the Jiaodong Peninsula region. **(B)** Bar chart showing reported HFRS cases by occupation. **(C)** Heat map showing the age distribution of reported HFRS cases by year. **(D)** Bar chart showing the age distribution of total reported HFRS cases.

Of the 1,492 cases of HFRS reported in the Jiaodong Peninsula region from 2019 to 2025, 1,075 were male (72.05%) and 417 were female (27.95%), with a male-to-female ratio of 2.58:1. The proportion of male cases was higher than that of female cases in every year (See Figure 5A). The occupational distribution was dominated by farmers (1,041 cases, 69.77%), followed by homemakers and unemployed individuals (147 cases, 9.79%), factory workers (111 cases, 7.44%), retired personnel (56 cases, 3.75%), and government officials and staff (34 cases, 2.28%), all other occupations accounted for less than 2% (See Figure 5B). The age distribution shows that cases were reported across all age groups from 2019 to 2025, with the majority concentrated in the 45–74 age group (1,079 cases, 72.32%), among these, the 55–59 age group accounted for the highest proportion (15.75%). followed by the 50–54 age group (15.42%) and the 65–69 age group (12.80%) (See Figures 5C,D). For detailed case data, (see Supplementary Table 1).

### 3.3 Analysis of incidence trends

**Figure 6:**
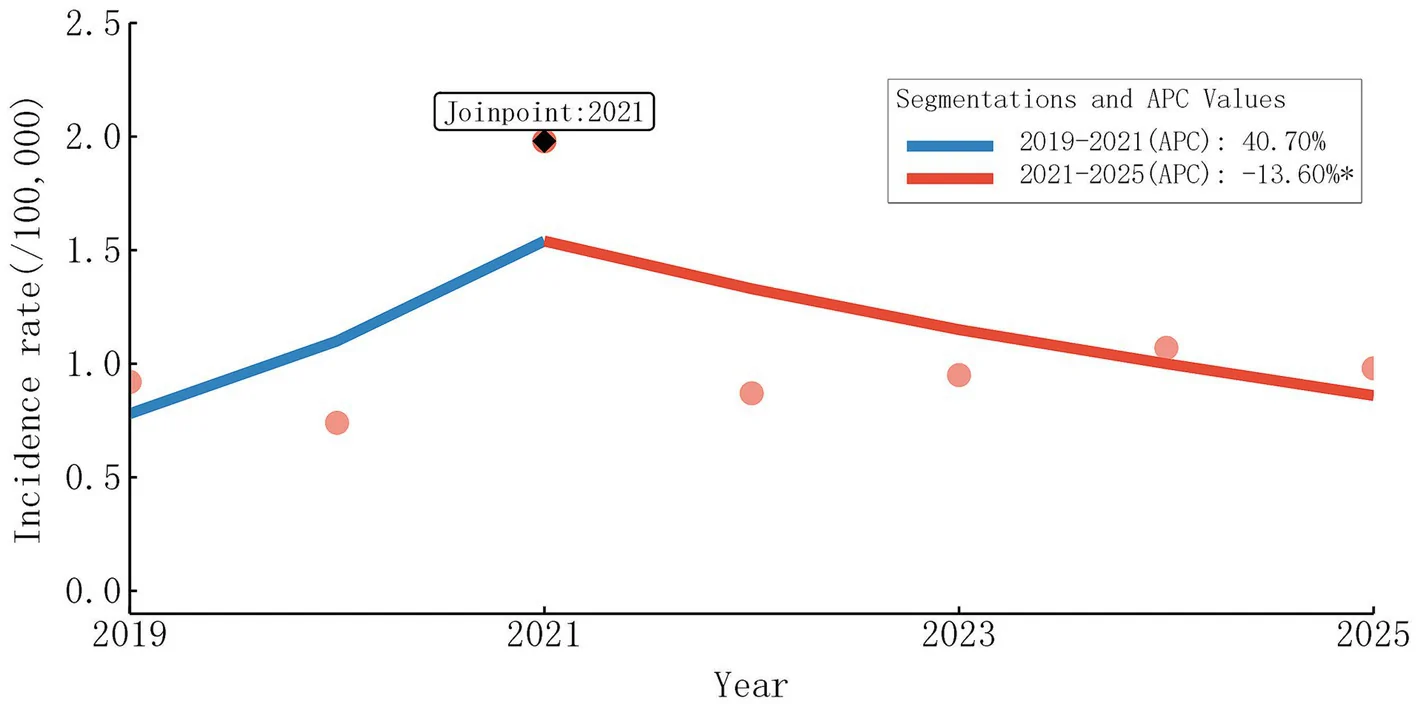
Trends in HFRS incidence rates in the Jiaodong Peninsula Region, 2019–2025.

Joinpoint regression analysis indicates that the incidence of HFRS in the Jiaodong Peninsula region from 2019 to 2025 followed an upward trend initially, followed by a decline, with a turning point occurring in 2021. From 2019 to 2021, the annual percentage change (APC) in incidence was 40.70% (95% CI: −15.72 to 134.88%, *p* = 0.103), and the upward trend was not statistically significant. From 2021 to 2025, the incidence rate showed a downward trend, with an APC of −13.60% (95% CI: −25.55 to 0.26%, *p* = 0.052), this downward trend reached marginal significance. The incidence rate reached its peak during the study period in 2021 (1.981 per 100,000), representing a significant increase of 168.1% compared to 2020 (0.739 per 100,000) (see Figure 6).

### 3.4 Spatial autocorrelation analysis

#### 3.4.1 Global spatial autocorrelation analysis

The results of global spatial autocorrelation analysis show that the Moran’s I index for the annual incidence of HFRS in the Jiaodong Peninsula region from 2019 to 2025 was positive in all years, with values of 0.205, 0.228, 0.438, 0.189, 0.433, 0.288, and 0.372. Except for 2019 (*p* = 0.052) and 2022 (*p* = 0.059), which showed no statistical significance, the remaining five years all exhibited significant spatial clustering (*p* < 0.05) with the strongest spatial clustering observed in 2021 (Moran’s I = 0.438, Z = 3.900, *p* = 0.001), followed by 2023 (Moran’s I = 0.433, Z = 3.710, *p* = 0.001). This indicates that HFRS incidence in the Jiaodong Peninsula region showed significant spatial positive correlation in most years, exhibiting a clustering pattern (See Table 1).

**Table 1:** Results of the global spatial autocorrelation analysis of HFRS data for the Jiaodong Peninsula region, 2019–2025.

Year	Moran’ I	Z score	*p*	Distribution status
2019	0.205	1.945	0.052	Random
2020	0.228	2.208	0.027	Cluster
2021	0.438	3.900	0.001	Cluster
2022	0.189	1.886	0.059	Random
2023	0.433	3.710	0.001	Cluster
2024	0.288	2.918	0.004	Cluster
2025	0.372	3.273	0.001	Cluster

#### 3.4.2 Analysis of local spatial autocorrelation

**Figure 7:**
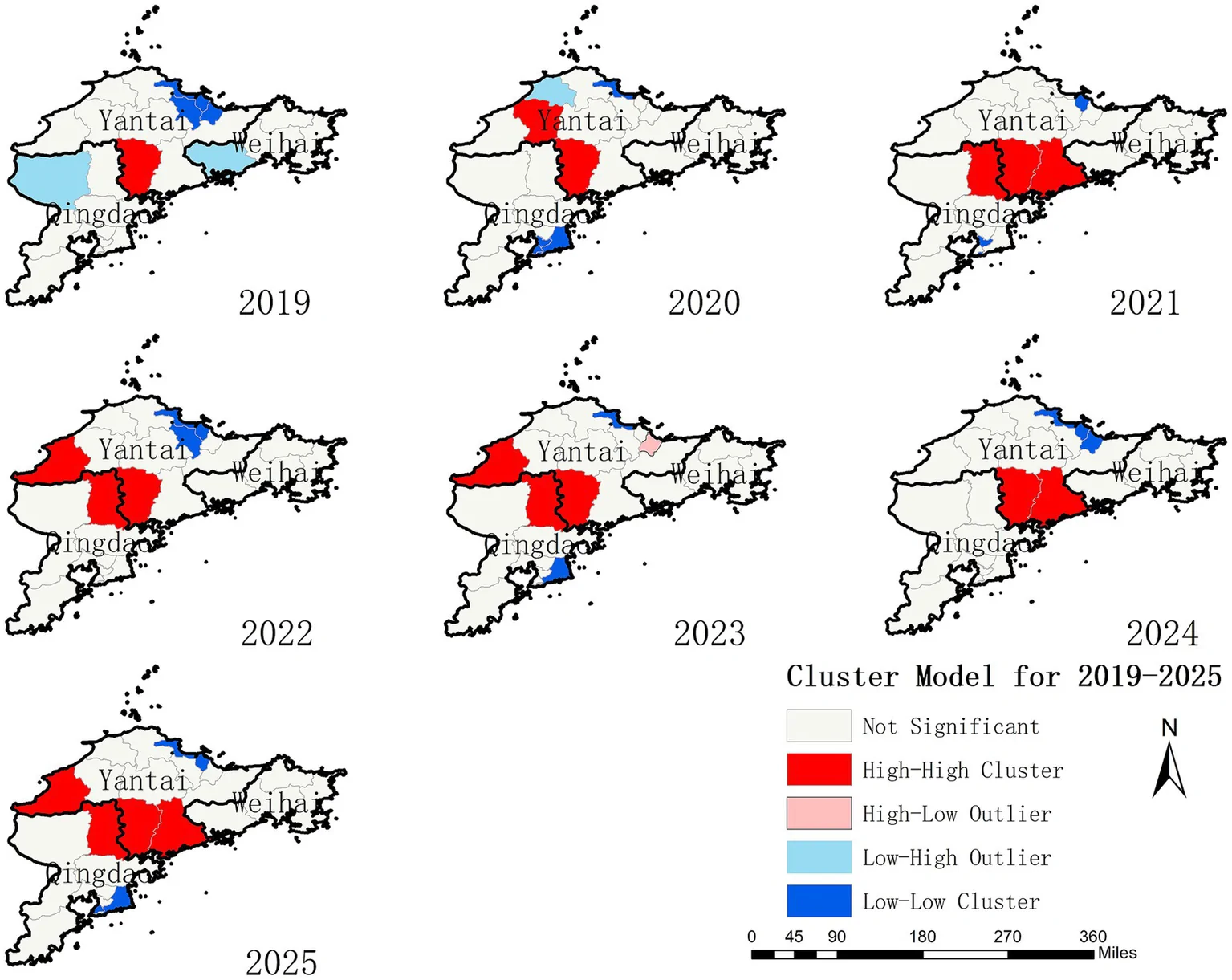
Analysis of local spatial autocorrelation of HFRS in the Jiaodong Peninsula Region, 2019–2025.

The spatial autocorrelation analysis map shows that the spatial distribution of high-high cluster zones (hotspots) for HFRS in the Jiaodong Peninsula region remained relatively stable from 2019 to 2025, primarily concentrated in the central and western parts of Yantai City (the Laiyang, Haiyang, and Laizhou areas) and the northern part of Qingdao City (the Laixi area). The LISA tests for high-high clusters in each year were statistically significant (*p* < 0.05). Among these, the high-high clusters in 2025 covered the largest area, with a total of four clusters: Laixi City, Qingdao (Local Moran’s I = 0.82, Z = 1.86, *p* = 0.05), Laiyang City, Yantai (Local Moran’s I = 1.96, Z = 2.59, *p* = 0.008), Laizhou City, Yantai (Local Moran’s I = 3.46, Z = 1.93, *p* = 0.046), and Haiyang City, Yantai (Local Moran’s I = 6.27, Z = 2.06, *p* = 0.038). In contrast, low-low clustering zones (cold spots) are relatively stable and distributed along the northern coast of Yantai (in the Penglai and Longkou areas) and in the southeastern part of Qingdao (in the Laoshan and Shinan areas). The clustering patterns exhibit dynamic evolution from year to year, with low-high clusters (outliers) appearing in 2019 and 2020. The low-high clusters in 2019 covered the widest area, suggesting an anomalous clustering phenomenon in that year, where a relatively large number of isolated counties and districts with high incidence rates were surrounded by areas with low incidence rates. High-low clustering zones appeared only in 2023. The extent of high-high clustering zones remained relatively stable from 2019 to 2024, but expanded significantly in 2025, indicating a trend toward the expansion of HFRS hotspots (see Figure 7).

### 3.5 Spatio-temporal cluster analysis

**Figure 8:**
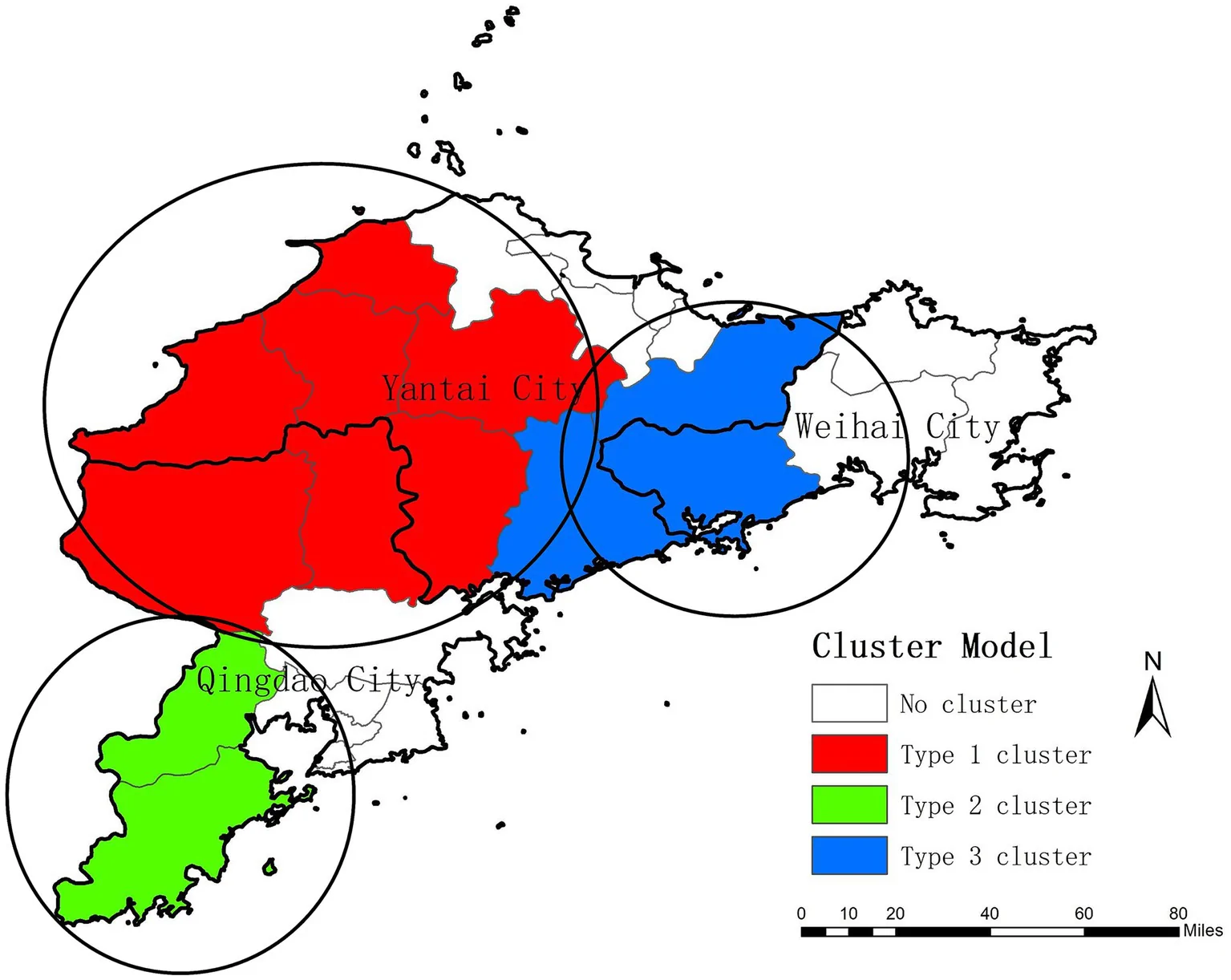
Spatiotemporal cluster analysis of HFRS in the Jiaodong Peninsula Region, 2019–2025.

The results of the spatiotemporal scan analysis (with the spatial window set to 30% of the total population in the study area and the temporal window set to 30% of the study period) show that there were three statistically significant spatiotemporal clusters of HFRS in the Jiaodong Peninsula region between 2019 and 2025, all of which occurred during the fall and winter of 2021 (see Figure 8). Type 1 cluster spanned seven counties (cities and districts), primarily distributed across five counties and districts in the inland and coastal areas of central and western Yantai (Laizhou City, Zhaoyuan City, Longkou City, Laiyang City, and Qixia City) and two counties/districts in northern Qingdao (Pingdu City and Laixi City). The time range was November 1, 2021, to December 31, 2021, with a scanning radius of 80.54 km. The actual number of reported cases in the region was 118, while the expected number was 9.39 (RR = 13.56, Log-Likelihood Ratio (LLR) = 194.16, *p* < 0.01), making it the core hotspot of the outbreak. Type 2 cluster spanned two counties (cities and districts), primarily located in Jiaozhou District and Huangdao District along the western and southwestern coast of Qingdao. The time range is from October 1, 2021, to December 31, 2021, with a scan radius of 42.85 km. The actual number of reported cases in the region was 66, and the expected number of cases was 7.84 (RR = 8.76, LLR = 83.67, *p* < 0.01). Type 3 cluster spanned three counties (cities and districts), located in Muping District in eastern Yantai, Haiyang City in southern Yantai, and Rushan City in western Weihai. The time range was from October 1, 2021, to December 31, 2021, with a scanning radius of 39.02 km. The actual number of reported cases in the region was 48, while the expected number of cases was 4.06 (RR = 12.17, LLR = 75.24, *p* < 0.01) (See Table 2).

**Table 2:** Results of the spatiotemporal cluster analysis of HFRS in the Jiaodong Peninsula Region, 2019–2025.

Cluster model	Scan radius (km)	Time range	Actual number of cases	Expected number of cases	Coverage	LLR	*p*	RR
Type 1 cluster	80.54	2021.11.01–2021.12.31	118	9.39	7	194.16	<0.01	13.56
Type 2 cluster	42.85	2021.10.01–2021.12.31	66	7.84	2	83.67	<0.01	8.76
Type 3 cluster	39.02	2021.10.01–2021.12.31	48	4.06	3	75.24	<0.01	12.17

### 3.6 Analysis of geographically and temporally weighted regression models

#### 3.6.1 Diagnosis of multicollinearity

This study selected the following meteorological variables: air temperature (°C), precipitation (mm), relative humidity (%), total solar radiation (down, MJ/m^2^), sea-level pressure (hPa), and wind speed (m/s). The results of the multicollinearity test show that the VIF for each variable is less than 10, indicating that there is no multicollinearity issue among these variables (see Table 3).

**Table 3:** VIF index for meteorological factors.

Meteorological factors	VIF
Air temperature(°C)	1.64
Precipitation (mm)	1.98
Relative humidity (%)	4.17
Total solar radiation (down, MJ/m^2^)	1.89
Sea-level pressure(hPa)	1.58
Wind speed (m/s)	3.12

#### 3.6.2 Comparison of model fitting performance

In this study, we constructed an OLS global model, a GWR spatial model, and a GTWR spatiotemporal geographically weighted regression model to compare their explanatory power regarding the spatiotemporal variability of HFRS incidence. The results showed that the OLS model (R^2^ = 0.23, adjusted R^2^ = 0.20) had limited explanatory power for HFRS incidence, suggesting that global models struggle to capture the spatial heterogeneity of the disease. After incorporating spatial weights into the GWR model, the R^2^ value was 0.28, confirming that there is significant spatial non-stationarity in HFRS incidence. The GTWR model incorporates both spatiotemporal weight matrices, with a coefficient of determination (R^2^) of 0.54 and an adjusted R^2^ of 0.43. Both values are significantly higher than those of the GWR and OLS models, indicating that the inclusion of the temporal dimension significantly enhances the model’s ability to capture the spatiotemporal patterns of HFRS and yields the best overall fit. When the AICc difference exceeds 3, the model with the lower value can be considered superior; the GTWR model has an AICc value of 172.30, which is the lowest among the three. Overall, the GTWR model performed best in balancing model complexity and goodness of fit, making it the most suitable for revealing the spatio-temporal heterogeneity of HFRS incidence on the Jiaodong Peninsula from 2019 to 2025 and its meteorological drivers (see Table 4).

**Table 4:** Comparison of overall fitting performance among OLS, GWR, and GTWR models.

Indicators	OLS	GWR	GTWR
R^2^	0.23	0.28	0.54
Adjusted R^2^	0.20	0.20	0.43
AICc	194.43	209.44	172.30

AICc, Corrected Akaike Information Criterion; OLS, ordinary least squares; GRW, geographically weighted regression; GTWR, geographically and temporally weighted regression (R^2^ and Adjusted R^2^) were calculated on the entire training set.

#### 3.6.3 GTWR model results

**Figure 9:**
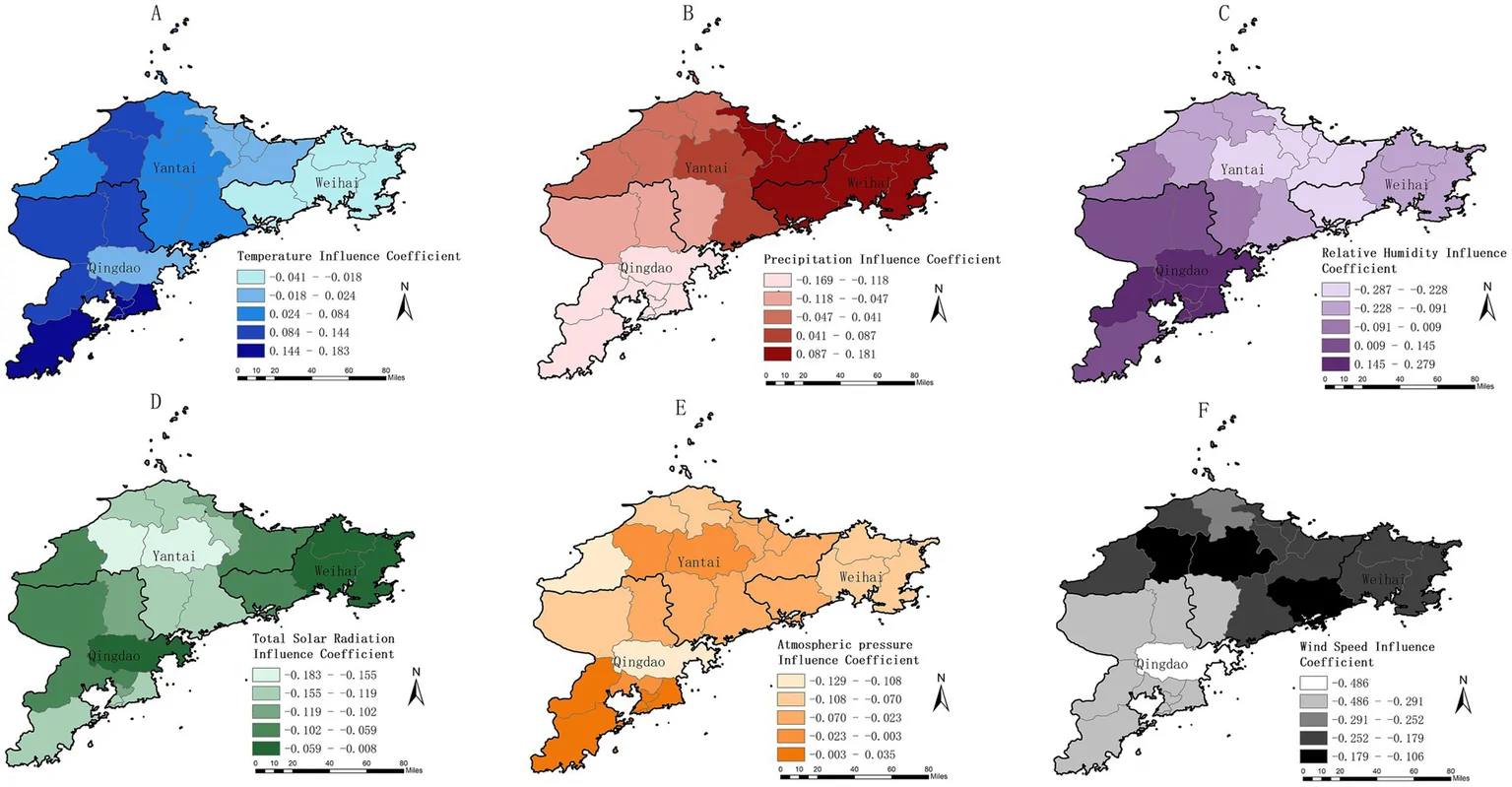
Spatial distribution of GTWR regression coefficients for meteorological factors affecting the risk of HFRS incidence in the Jiaodong Peninsula Region, 2019–2025. **(A)** influence coefficient for air temperature; **(B)** influence coefficient for precipitation; **(C)** influence coefficient for relative humidity; **(D)** influence coefficient for total solar radiation; **(E)** influence coefficient for atmospheric pressure; **(F)** influence coefficient plot for wind speed.

Based on the annual local regression coefficients for 2019–2025 generated by the GTWR model, the 7-year average coefficients for each meteorological factor were extracted and visualized spatially to illustrate the long-term average effects of these factors on the incidence of HFRS. As shown in Figure 9A, the effect of temperature on HFRS incidence exhibits significant spatiotemporal heterogeneity, with regression coefficients ranging from −0.041 to 0.183. Overall, a spatial pattern of “strong in the west, weak in the east; high in the south, low in the north” emerges. The temperature impact coefficient is positive in most regions, indicating that rising temperatures are generally positively correlated with the risk of HFRS outbreaks; only in the eastern region is the coefficient weakly negative. In particular, the darkest areas are in southern Qingdao, indicating that temperature has the strongest positive influence on HFRS incidence. Figure 9B shows that precipitation generally exhibits an “eastern high, western low” distribution pattern, indicating that precipitation in Weihai and the eastern part of Yantai is positively correlated with the risk of HFRS, whereas precipitation in Qingdao is negatively correlated with the risk of HFRS; in other words, increased precipitation has a certain inhibitory effect on HFRS incidence. Figure 9C shows that in Qingdao, the higher the relative humidity, the higher the risk of HFRS; in contrast, in eastern Yantai and Weihai, rising humidity has a suppressing effect on the incidence of the disease. Figure 9D shows that the regression coefficients for total solar radiation range from −0.183 to −0.008, with all regions exhibiting negative values. This indicates that solar radiation exerts a negative inhibitory effect on the incidence of HFRS across the entire Jiaodong Peninsula, with no regions showing a positive effect. As shown in Figure 9E, atmospheric pressure generally exerts a negative influence, with the strongest suppressing effect observed in western Yantai, while a very weak positive influence is evident in localized areas along the southern coast of Qingdao. Figure 9F shows that the regression coefficients for wind speed range from −0.486 to −0.106, with all values being negative. This indicates that wind speed exerts a significant negative inhibitory effect on HFRS incidence across the entire Jiaodong Peninsula, and is one of the variables with the strongest inhibitory effect; the intensity of this inhibitory effect gradually weakens from southern Qingdao to northern Yantai and eastern Weihai.

## 4 Discussion

Hemorrhagic fever with renal syndrome (HFRS) is a naturally occurring infectious disease caused by Hantaviruses. It can be transmitted through various routes, including respiratory and contact transmission, and poses a serious threat to human health. It has become one of the Category B infectious diseases prioritized for prevention and control in China (2). As one of the key regions in China where HFRS is prevalent, the Jiaodong Peninsula’s unique geographical environment and climatic conditions may influence the distribution and clustering patterns of HFRS. This study conducts a descriptive epidemiological and spatiotemporal clustering analysis of HFRS in the Jiaodong Peninsula region from 2019 to 2025. Using R, ArcGIS, SaTScan, and Joinpoint regression analysis software, the data are systematically analyzed and visualized. The aim is to clarify the temporal, regional, and population distribution patterns of HFRS in this region, as well as its spatiotemporal clustering characteristics, thereby providing crucial spatiotemporal epidemiological support for the development of refined prevention and control strategies for HFRS in the region.

The results show that the incidence of HFRS in the Jiaodong Peninsula fluctuated between 2019 and 2025, with 2021 marking a peak year, when the annual incidence reached 0.915 per 100,000. This may be attributed to meteorological conditions (temperature, precipitation, and humidity) remaining within the high-risk range for HFRS from 2019 to 2021, rodent population density increased year by year, and the virus prevalence rate continued to rise (10). Additionally, early epidemic monitoring data lacked sufficient accuracy in predicting the autumn-winter peak, key populations were either unvaccinated or incompletely vaccinated (25). However, the specific reasons for the resurgence of cases still require comprehensive analysis based on multiple data sources, including epidemiological investigations and serological testing. After 2021, the incidence of HFRS declined as comprehensive prevention and control measures-including rodent control, and environmental management-were implemented (26). In addition, HFRS exhibits a distinct seasonal pattern, with outbreaks peaking primarily during the fall and winter months (October through January of the following year). Cases reported during this period account for 70.58% of the total, making it a critical window for HFRS prevention and control. This pattern is consistent with the findings of most studies in the literature (27). This may be related to the population structure of the host species in the region, the dominant rodent species in the Jiaodong Peninsula is the black-striped field mouse. This species does not hibernate and tends to migrate from habitats such as farmlands and hills to human settlements and grain storage areas during the fall and winter, resulting in a significant increase in opportunities for contact with humans (28).

In terms of regional distribution, areas with high incidence of HFRS are primarily concentrated in central and western Yantai and northern Qingdao, exhibiting a distinct spatial clustering. Geographically, these regions are characterized by low mountains and hills, farmland, and proximity to the coast, with a humid climate that provides ideal habitats for the breeding and reproduction of HFRS vectors (10). At the same time, agricultural activities are concentrated in this area, and farmers frequently work in the fields. This is consistent with the findings of Lian Yanyan et al. (29), who concluded that areas with high incidence of HFRS are predominantly concentrated in regions with developed agriculture and abundant rodent habitats. In terms of population distribution, the high-risk groups for HFRS are primarily men, farmers, and individuals aged 45–74. Farmers account for as much as 69.77% of the population in the Jiaodong Peninsula region, a proportion significantly higher than that of other occupational groups. Furthermore, the Backward-Step Regression (BRT) risk prediction model developed by Shandong Province confirms that cropland (relative contribution rate of 9.98%), forested land (8.71%), and rural settlements (9.25%) are key landscape factors influencing the prevalence of HFRS. The areas identified in this study as having high-high clustering are the primary production regions for commercial fruit crops such as apples and cherries, where farmers have greater opportunities for contact with rodents, for a variety of reasons, farming has become a high-risk occupation (9). This is also highly correlated with the transmission routes of HFRS and the characteristics of population exposure risk, and is consistent with the overall epidemiological patterns of HFRS nationwide. One possible reason why men are at higher risk is that a higher proportion of men than women engage in outdoor work and agricultural labor, resulting in greater opportunities for contact with rodents and exposure to Hantaviruses. This aligns with the conclusions of the “Expert Consensus on the Prevention and Control of Hemorrhagic Fever with Renal Syndrome (2021 Edition)” (17), which identifies middle-aged and older men as high-risk groups and reports a male-to-female incidence ratio of approximately 3:1. It is also largely consistent with the results of HFRS surveillance in Qingdao, which showed a male-to-female incidence ratio of 2.88:1 (30). The results of the join-point regression analysis indicate that the incidence of HFRS in the Jiaodong Peninsula region from 2019 to 2025 generally followed a fluctuating trend of first rising and then declining, The year 2021 served as the turning point in incidence throughout the study period, reflecting the dynamic evolution of HFRS epidemics in this region. This finding is consistent with relevant studies in the Weifang area. Zhang et al. reported a rebound in HFRS cases in 2021, aligning with the conclusion that 2020 was the year with the lowest incidence (31). The increase observed between 2019 and 2021 was not statistically significant (*p* = 0.103). This is thought to be related to factors such as the relatively short study period, data fluctuations during the early stages of the pandemic, and changes in case reporting, therefore, it is not yet possible to conclude that a clear upward trend exists (32). After 2021, the incidence rate showed a marginally significant decline (APC = −13.60%, *p* = 0.052), suggesting that disease control measures, environmental changes, and shifts in host animal density may have contributed to the control efforts. The incidence rate has been gradually declining, which has important public health implications (33).

A global spatial autocorrelation analysis revealed that HFRS cases in the counties (cities and districts) of the Jiaodong Peninsula exhibited spatial clustering patterns from 2019 to 2025. With the exception of 2019 and 2022, significant positive spatial correlation was observed in all other years, and the intensity of clustering showed a trend of changing in tandem with the peak incidence of HFRS. The persistent occurrence of HFRS clusters in the region may be closely related to local topography and agricultural production patterns. Areas with high HFRS incidence are predominantly found on gentle slopes of low-altitude hills, where vegetation coverage is high and the terrain is relatively enclosed; agricultural lands such as farm fields and orchards are all classified as high-risk landscapes. Agricultural activities are frequent throughout the year in these areas, and people spend extended periods working outdoors. This environment is conducive to the habitation and reproduction of rodent reservoir hosts, thereby ensuring that the source of infection remains stable over the long term (34). At the same time, resources for routine infectious disease prevention and control at the grassroots level may be prioritized for COVID-19 prevention and control, which could weaken efforts in routine rodent control and HFRS surveillance (35). For years such as 2021 and 2023, which saw a high concentration of cases in specific regions, comprehensive prevention and control measures-including rodent control should be prioritized. Conversely, for years such as 2019 and 2022, which saw a lower concentration of cases, the focus should be on surveillance and early warning systems to prevent the spread of localized clusters. Analysis of local spatial autocorrelation further indicates that HFRS cases in the Jiaodong Peninsula region are showing a trend of spreading westward, while the spatial distribution of high-high and low-low cluster zones remains relatively stable overall. High-high cluster zones are consistently distributed in the central and western parts of Yantai City and the northern part of Qingdao City. The primary reason is that the region features a landscape of low mountains and hills, with commercial orchards (such as apple and cherry orchards) and dryland farmland constituting the dominant landscape types. The geographical and ecological conditions align with the habitat requirements of the primary host animals, and between 2019 and 2025, there were no significant changes in the region’s patterns of cropland, forested areas, and water systems. Additionally, high-risk groups such as farmers frequently engage in outdoor work, significantly increasing opportunities for human-rodent contact and thereby forming stable high-incidence cluster zones (36, 37). Li et al.’s study also confirmed that farmland, forests, grasslands, and orchards provide natural habitats for rodents (38). Low-low risk areas are relatively stable and concentrated in the northern coastal region of Yantai City and the southeastern part of Qingdao City. This region consists primarily of coastal urban areas or suburban zones with high levels of urbanization, comprehensive rodent control measures, a systematic waste management system and relatively good sanitary conditions. Natural habitats such as farmland have been largely replaced by urban construction land, resulting in a severe compression of the host animals’ living space. Population densities are significantly lower than in inland agricultural areas, thereby forming HFRS “cold spots.” This finding is consistent with research conclusions indicating that urbanization can reduce the risk of HFRS by altering the habitats of host animals, and it also corroborates the spatial distribution pattern of low HFRS incidence in eastern coastal cities of China (6, 39).

The results of the spatiotemporal scanning analysis show that three statistically significant spatiotemporal clusters of HFRS were identified in the Jiaodong Peninsula region between 2019 and 2025, all of which were concentrated in the fall and winter of 2021. This finding is highly consistent with the conclusion from the previous spatial autocorrelation analysis that “2021 was the peak year for HFRS incidence and the year with the strongest spatial clustering,” further confirming that the fall and winter of 2021 was a critical period for the HFRS outbreak in the Jiaodong Peninsula region, consistent with the spatial clustering of cases resulting from extreme precipitation and high temperatures during that time and in that region, and underscores the importance of epidemic prevention and control during that period and in that area. These three clusters, identified as the epicenters of the outbreak, are characterized by a wide geographical coverage and a high concentration of cases. The reasons for their high incidence are closely linked to the geographical and ecological characteristics of the low mountains and hills in this region and the population’s risk of exposure, consistent with the findings of previous spatiotemporal scanning analyses of HFRS in Beijing, which concluded that “high-incidence clusters are predominantly concentrated in areas suitable for the habitat of reservoir animals and where the risk of human exposure is relatively high” (6, 40). For the three spatio-temporal clusters and the high-risk areas, it is essential to significantly increase the allocation of prevention and control resources and strengthen intervention measures-such as monitoring of reservoir animals and vaccination of high-risk populations-to prevent concentrated outbreaks or cross-regional spread of the disease. The results of the spatio-temporally weighted regression (GTWR) model analysis indicate that the GTWR model, which incorporates spatio-temporal dimensions, is more effective in characterizing the spatio-temporal heterogeneity of HFRS incidence. This finding is consistent with previous studies, which suggest that the GTWR model can more accurately reveal the dynamic mechanisms by which meteorological factors influence HFRS incidence through the construction of a spatio-temporal weight matrix. It should be noted that this study used 7-year average coefficients for spatial visualization, which may obscure interannual fluctuations; future studies could present the spatiotemporal evolution of these coefficients on an annual basis (13, 23). The effect of temperature on HFRS incidence in the Jiaodong Peninsula exhibits a spatial pattern characterized by “higher incidence in the west than in the east, and higher in the south than in the north.” The regression coefficients were positive in most regions, indicating that rising temperatures increase the risk of HFRS outbreaks; this finding is consistent with domestic studies. Rising temperatures can promote the reproduction and activity of host animals, thereby increasing opportunities for human-rodent contact (31). The impact of precipitation varies by region. In Weihai and eastern Yantai, precipitation is positively correlated with the risk of disease outbreaks; increased precipitation promotes vegetation growth, providing food sources for rodents and increasing their population density. This pattern has also been confirmed by studies in the Xuancheng area, whereas in the Qingdao area, precipitation has a negative effect (41). Appropriate humidity levels can prolong the survival of Hantavirus *in vitro*, and the humidity conditions in Qingdao are more conducive to HFRS outbreaks; however, both excessively high and low humidity levels can inhibit viral activity (10). Overall, atmospheric pressure has a negative effect, it primarily affects animal activity through its impact on precipitation. Low pressure is often accompanied by changes in temperature and humidity, which indirectly influence the activity rhythms of rodents. However, the underlying mechanism still requires verification through host behavioral experiments (42). Total solar radiation and wind speed both had a significant negative inhibitory effect on the incidence rate across the entire study area, with wind speed having the strongest effect. Ultraviolet radiation can inactivate Hantavirus, thereby reducing its transmissibility and survival time (43). Strong winds can alter the dispersion and deposition of viral aerosols and also suppress rodent activity outdoors, thereby indirectly reducing the risk of human-rodent contact; however, the direct inhibitory effect of strong winds on rodent activity still requires verification through host behavioral experiments (44).

This study also has certain limitations: (1) No correlation analysis was conducted on factors such as Hantavirus genotypes, vaccination coverage, and rodent population data, these could be explored in greater depth in future studies. (2) Differences in land area, population density, and spatial patterns across counties and districts may introduce bias into the Moran’s I index and spatiotemporal analysis results, future studies could employ data at the township, subdistrict, or grid level to conduct more detailed analyses. (3) The study period covers the COVID-19 pandemic from 2020 to 2022, during which public health resources, residents’ healthcare-seeking behavior, and infectious disease surveillance were all affected, which may have introduced confounding factors into the HFRS data. (4) The counties bordering the study area are prone to the spread of the disease, as well as the movement of hosts and people, to surrounding regions. In future work, the scope of the study will be expanded to cover the entire province of Shandong to further validate the robustness of the findings.

## 5 Conclusion

In summary, the incidence of HFRS in the Jiaodong Peninsula region showed an overall downward trend from 2019 to 2025, although there remains a risk of a resurgence, 2021 marked the peak year for HFRS incidence. HFRS exhibits distinct seasonal patterns, with middle-aged and older male farmers constituting the high-risk population. The disease also shows significant spatial clustering in the region, primarily concentrated in central and western Yantai and northern Qingdao. Furthermore, the impact of meteorological factors on HFRS incidence exhibits significant spatiotemporal heterogeneity. In the future, we should integrate the spatiotemporal epidemiological characteristics of HFRS with meteorological drivers, strengthen surveillance, early warning, and comprehensive prevention and control measures in key areas and among high-risk populations. This will provide a scientific basis for targeted interventions among high-risk groups and early outbreak warnings, thereby reducing the risk of clustered outbreaks.

## Data Availability

The data analyzed in this study is subject to the following licenses/restrictions: raw data cannot be made publicly available due to confidentiality and privacy. The data includes sensitive information that could identify patients or compromise their privacy. Moreover, all relevant data have been included in the manuscript and accompanying figures. Readers interested in this study may contact the corresponding author via email if they wish to learn more about the research. Requests to access these datasets should be directed to Qing Duan, Email: sdcdcdq@163.com.
