# Supplementary material for: Epidemiological characteristics and spatiotemporal heterogeneity of hemorrhagic fever with renal syndrome in the Jiaodong Peninsula region, China, 2019–2025
Source: Front Public Health. 2026 Aug 20;14:1868358. doi: 10.3389/fpubh.2026.1868358 (PMC13538979; doi:10.3389/fpubh.2026.1868358)
Supplement: Supplementary file 1 [file Data_Sheet_1.PDF]

Supplementary Table 1 Distribution of HFRS Patients in the Jiaodong Peninsula Region, 2019-2025

Category		Number of cases	Proportion (%)
Gender	male	1075	72.05
	female	417	27.95
Age	0-	1	0.07
	5-	1	0.07
	10-	5	0.34
	15-	25	1.68
	20-	29	1.94
	25-	35	2.35
	30-	61	4.09
	35-	87	5.83
	40-	87	5.83
	45-	133	8.91
	50-	230	15.42
	55-	235	15.75
	60-	175	11.73
	65-	191	12.8
	70-	115	7.71
	≥75	82	5.5
Occupation	Nursery children	0	0
	Preschool children	1	0.07
	Students	25	1.68
	Teachers	1	0.07
	Childcare workers	0	0
	Catering & food workers	12	0.8
	Public venue service workers	1	0.07
	Commercial service workers	17	1.14
	Healthcare workers	0	0
	Factory workers	111	7.44
	Migrant laborers	17	1.14
	Farmers	1041	69.77
	Herdsmen	0	0
	Fishermen	2	0.13
	Seafarers & long-haul drivers	1	0.07
	Civil servants	34	2.28
	Retirees	56	3.75
	Homemakers & unemployed	147	9.79
	Unknown	11	0.74
	Others	16	1.07
